# Evaluability assessment of “growing healthy communities,” a mini-grant program to improve access to healthy foods and places for physical activity

**DOI:** 10.1186/s12889-019-7156-8

**Published:** 2019-06-20

**Authors:** Christiaan G. Abildso, Angela Dyer, Shay M. Daily, Thomas K. Bias

**Affiliations:** 1 West Virginia University School of Public Health, 64 Medical Center Drive, PO Box 9190, Morgantown, WV 26506-9190 USA; 2 University of Southern Maine, Portland, ME 04104 USA

**Keywords:** Public health, Built environment, Community financing, Mini-grants, Rural development

## Abstract

**Background:**

Mini-grants have been used to stimulate multisector collaboration in support of public health initiatives by funding non-traditional partners, such as economic development organizations. Such mini-grants have the potential to increase access to healthy foods and places for physical activity through built environment change, especially in small and rural towns in the United States. Although a promising practice, few mini-grant evaluations have been done. Therefore, our purpose was to conduct an Evaluability Assessment (EA), which is a process that can help promising programs that lack evidence advance toward full-scale evaluation. Specifically, we conducted an Evaluability Assessment of a statewide mini-grant program, called “Growing Healthy Communities” (GHC), to determine if this program was ready for evaluation and identify any changes needed for future implementation and evaluation that could also inform similar programs.

**Methods:**

Telephone interviews with directors of six past mini-grant recipient organizations were conducted to assess implementation and evaluability. The six interviews were split equally among agencies receiving funding for food-oriented projects and physical activity-oriented projects. Within- and cross-case thematic analyses of interview transcripts were conducted.

**Results:**

*Organizational capacity* was a universal theme, reflecting other key themes (described in detail in the manuscript) that affected program implementation and evaluation, including *collaboration*, *limited time* and *measurement integration*. *Conclusions*. The EA process provided pilot data that suggest that other state, regional, and national funders should provide centralized assistance for data collection and evaluation from the outset of a mini-grant award program.

**Electronic supplementary material:**

The online version of this article (10.1186/s12889-019-7156-8) contains supplementary material, which is available to authorized users.

## Background

Over the past two decades, mini-grants have been used for community-based public health initiatives, especially in rural areas, to address priority topics of national interest. As noted by Bobbitt-Cooke [1], this was largely in response to United States Surgeon General Dr. David Satcher’s challenge in the early 2000s to incentivize community-based organizations to implement activities in support of *Healthy People 2010*. The purpose of mini-grants (sometimes called “micro-grants”) is to support government entities, non-profit organizations, small businesses, or other interest groups to stimulate quick, positive changes in communities. These are possible to implement when awarded to trusted, community-based organizations that can facilitate multi-disciplinary solutions to problems because of strong existing collaborations, communication, and engagement among collaborators [2].

Extant literature highlights the wide range of public health initiatives supported by mini-grants, including physical activity [3–9], healthy eating [5, 7, 8, 10], childhood wellbeing [11], and other objectives outlined in *Healthy People 2020* [1, 12]. For such public health initiatives, mini-grant funding varies substantially per funded project, from as low as $40 to as much as $50,000 [7]. Early evaluations of this approach focused, necessarily, on “process,” specifically the organizational administrative processes and partnerships needed to successfully administer the funding and implement the community-based activities [1, 3, 12]. In aggregate, this limited body of work highlights the promise of mini-grants for quick and inexpensive implementation of projects to affect targeted health behaviors.

Mini-grant programs are particularly enticing for rural areas to develop health-focused partnerships among organizations with diverse missions but are untested with economic development entities. The limited literature suggests that rural mini-grant recipients, such as schools, churches, neighborhoods, and youth groups, do not necessarily have to have a primary focus on health but can adapt and collaborate to address priority health topics at the community-level [1, 8, 12]. Specifically, child care centers [13], community-based organizations [14], and faith-based organizations [5] have demonstrated the capability to improve access to healthy food and places for physical activity through policy, systems, and environment change within their own organizational contexts (e.g., healthier potluck after church service). Mini-grants are attractive for smaller organizations because funding is primarily or exclusively directed to implementation with little to no funding redirected to outside organizations for evaluation [1]. This focus points to a key deficiency in developing the evidence-base for this type of work, however, because of the lack of evaluation expertise in these community organizations and the lack of understanding of the impact of small projects that can be funded by the limited resources of mini-grants. Further developing this evidence is critical to understanding the potential of mini-grants in addressing the vast health disparities experienced in rural America.

Main Street America (MSA; https://www.mainstreet.org/) is a wide reaching national economic development program in historic downtowns, including rural ones, that may be perfectly positioned to receive and implement mini-grant funding for health initiatives. MSA is a program of the National Main Street Center, a subsidiary of the National Trust for Historic Preservation, operating in over 1600 communities across the United States providing education, training, technical assistance, and resources to a network of state, county, and local organizations. The MSA approach focuses on strengthening or revitalizing downtowns or commercial districts through four strategies: Economic Vitality, Design, Promotion, and Organization. This approach shares much with the ecological view of public health that suggests that providing safe, convenient access to places for physical activity and healthy eating is a key determinant of these critical health behaviors [15]. Historic downtowns are a place for such access, especially in rural areas, where economic development and access to healthy places can be mutually reinforcing. While the idea of utilizing mini-grants to combine local economic development with efforts to improve public health in rural areas has great potential, evaluations of such attempts are limited and could benefit from pilot studies [11, 12] to determine if mini-grant programs are ready for full-scale evaluation. Thus, the purpose of this study was to conduct an Evaluability Assessment of a pilot mini-grant program to (a) determine if this program was ready for evaluation and (b) identify any changes needed for future implementation and evaluation that could also inform similar programs.

## Methods

### Intervention approach

The Growing Healthy Community (GHC) Collaborative Grant Program was established in 2013 by the Claude Worthington Benedum Foundation, the West Virginia (WV) Department of Health and Human Resources (DHHR), and the Main Street WV (MSWV) and WV Organization, Training, Revitalization, and Capacity (ON TRAC) programs. MSWV and ON TRAC are affiliated with MSA and housed within the state’s Development Office. Annually from 2013 to 2017, the Claude Worthington Benedum Foundation and the WV DHHR provided grant funding to the WV Development Office who then administered mini-grants of up to $25,000 each to awardees selected in a competitive process. All 13 MSWV and 11 WV ON TRAC organizations were eligible to apply. As of August 2017, over $600,000 had been awarded for 38 projects across 24 communities with the goal of bridging economic revitalization and health.

Funded projects generally focused on providing access to healthy foods and places for physical activity, while concurrently encouraging economic activity. Projects often reflected the recommendations of The Community Guide to Preventive Services *Creating or Improving Places for Physical Activity* [16] or the Centers for Disease Control and Prevention’s *Recommended Community Strategies and Measurements to Prevent Obesity in the United States* (“COCOMO” strategies) as adapted to rural settings [17]. Example projects include developing walkability or connectivity plans; building or enhancing local trails; expanding farmers markets or offering monetary incentives to use them; and promoting healthy living and exercise. Though promising, the few studies that have attempted to evaluate the link between economic and health measures of such approaches, have been unable to establish reliable measurement approaches for accurate comparisons [11, 12].

### Evaluability assessment

An EA – sometimes called “Exploratory Evaluation” or “Pre-Evaluation” – is a process that may precede a full-scale, time- and resource intensive evaluation of a policy, program, or practice to help address common evaluation challenges that often lead to null or inconclusive findings [18, 19]. These challenges include stakeholder disagreement, lack of understanding of logic or theory of change, unrealistic program goals, and/or unclear outcomes and measurement methods [18]. There is no single accepted model for conducting an EA, but some common elements exist across prior work [20, 21], including: reviewing program documentation; engaging stakeholders; identifying a guiding logic model or theory of change; and planning future evaluation design, priorities, and uses. The process has also been suggested as a method of identifying practice-based evidence to inform public health practice [22]. Typical outcomes of an EA include the facilitation of a deeper understanding among stakeholders about program implementation, development of a program logic model or theory of change, identification of needs for program implementation, determination of a program’s readiness for a full evaluation, and/or identification of potential program improvements [18, 21, 22].

A qualitative approach was utilized to conduct the EA for the GHC program. The ten EA steps from Smith’s model [21] were used chronologically, and are presented in the following paragraphs organized into three groups: Organization, Stakeholder Engagement, and Assessing Implementation/Making Recommendations (see Table 1). All data collection procedures and use of human subjects were approved by the West Virginia University Institutional Review Board (protocol # 1703490460).

**Table 1:** Activities conducted as part of an Evaluability Assessment of the West Virginia Growing Healthy Communities mini-grant program, 2017

Evaluability Assessment Step	Grouping of Steps	Activities
Step 1: Determine Purpose, Secure Commitment, and Identify Work Group Members	Organization	• Secured client agreement with MSWV and ON TRAC Director
Step 2: Define Boundaries of Program to be Studied		• Created syllabus, schedule of meetings, activities, and deliverables
Step 3: Identify and Analyze Program Documents		• Conducted document-, gray-, and scientific literature reviews
Step 4: Develop/Clarify Program Theory	Stakeholder Engagement	• Held in-person meeting with MSWV and ON TRAC directors
Step 5: Identify and Interview Stakeholders		• Conducted telephone interviews with two national Main Street stakeholders
Step 6: Describe Stakeholder Perceptions of Program		• Held in-person meeting with MSWV and ON TRAC directors, and WVDHHR and Claude Worthington Benedum Foundation representatives
Step 7: Identify Stakeholder Needs, Concerns, and Differences in Perceptions		• Conducted telephone interviews with staff from six local Main Street GHC mini-grant recipients
Step 8: Determine Plausibility of Program Model	Assessing Implementation / Making Recommendations	• Assessed scientific literature with results of interviews with state and national Main Street stakeholders
Step 9: Draw Conclusions and Make Recommendations		• Presented findings to MSWV and ON TRAC directors, and WVDHHR and Claude Worthington Benedum Foundation representatives in person
Step 10: Plan Specific Steps for Utilization of Evaluability Assessment Data		• Presented findings and led a logic model training with all MSWV and ON TRAC programs at state-level conference
		• Provided material for presentation by MSWV stakeholder at national Main Streets meeting.
		• Created a logic model toolkit for use in future GHC grant applications
		• Prepared a research manuscript and research briefs.

Note: *MSWV* Main Street West Virginia, *ON TRAC* West Virginia Organization, Training, Revitalization, and Capacity, *WVDHHR* West Virginia Department of Health and Human Resources

### Evaluability assessment steps 1–3: organization

The EA was conducted by the evaluation team from January to May of 2017 as part of a graduate level course on program evaluation under the instructor’s supervision. Prior to the semester, the Directors of the MSWV and ON TRAC programs agreed to serve as a “client” for whom the evaluation team would work (EA Step 1) and came to an agreement with the course instructor about activities, meeting schedules, and deliverables that were incorporated into the course syllabus (EA Step 2). The initial task completed by the evaluation team was a review of the mini-grant peer-reviewed literature and gray literature about the GHC program, MSWV and ON TRAC, and MSA (EA Step 3).

### Evaluability assessment steps 4–7: stakeholder engagement

Following the document review, a meeting was held with the MSWV and ON TRAC directors at which they presented all existing material about the GHC program history and implementation. The evaluation team worked with the directors to clarify program theory and implementation, identify potential outcomes from the evaluation team’s work, and develop a list of national-, state-, and local stakeholders. (EA Steps 4 and 5) Structured telephone interviews with two national-level stakeholders were then held to identify the broader context for the evaluation team’s work. (EA Step 6) Key findings from these stakeholder engagement activities and document reviews included the following:

1. Insufficient primary data from GHC projects existed to conduct an outcome evaluation;
2. There were no outcome measures from national datasets with local specificity that could be used to conduct an evaluation;
3. There were a limited number of outcome evaluations in the current mini-grant literature;
4. State and national stakeholders were interested in incorporating health as a key outcome metric along with the economic development outcomes

Subsequently, the evaluation team presented a proposal of activities to address these four needs during an in-person meeting with the MSWV and ON TRAC directors and representatives from the funding agencies of the GHC program (WVDHHR and the Claude Worthington Benedum Foundation). (EA Step 7).

### Evaluability assessment steps 8–10: assessing implementation/making recommendations

In Step 7, agreement was reached with the stakeholders for the evaluation team to conduct structured telephone interviews with directors of six past GHC recipient organizations to assess implementation (EA Step 8) and make recommendations (EA Step 9). The six sites were identified by the MSWV and ON TRAC directors as the “most successful” in project implementation based on the directors’ perceptions. The six interviews were split equally among agencies receiving funding for food-oriented projects and physical activity-oriented projects (see Table 2). Each interview was scheduled via email, at which time a cover letter was attached with information about the purpose of the evaluation and use of the information collected. All telephone interviews were conducted by one member of the evaluation team following a script that was developed specifically for this project and reviewed by key stakeholders prior to the interviews (Additional file 1). The script contained seven items designed to capture stakeholder capacity, program sustainability, successes, barriers, key health and economic outcomes, and recommendations for improvement of the program. Interviews lasted between 15 and 33 min (average 24:02; see Table 2) or until content saturation – lack of novel information – was met (interviewer determined). No incentives were issued to participants.

**Table 2:** Characteristics of Sites whose Directors Participated in Interviews, conducted as part of an Evaluability Assessment of the West Virginia Growing Healthy Communities mini-grant program, 2017

Site	Population^a^	Project Description	Grant Amount ($)	Interview Length (mm:ss)
Physical Activity-oriented Projects (PA)				
PA1	17,227	Create a downtown walking program, implement worksite wellness programs for downtown businesses, and create a health and wellness kiosk in the downtown.	$25,000	15:57
PA2	7094	Construct curbing for a path connecting downtown business district with historic attractions nearby.	$17,500	22:05
PA3	3252	Complete a water trail.	$25,000	27:28
Food-oriented Projects (F)				
F1	2939	Install a high-tunnel and community garden to encourage gardening among seniors, and implement farmers market vouchers for WIC participants.	$12,490	22:35
F2	1765	Develop a community garden.	$9250	24:24
F3	28,486	Conduct activities to support the opening of a year-round, indoor farmers market in downtown.	$25,000	32:06

^a^ 2010 US Census (www.census.gov)

Note: a high-tunnel (or “hoophouse”) is a type of unheated greenhouse that comes in many sizes that is used to extend the growing season in farming

The final EA Step (Planning for use of EA data) was built in to each of the stakeholder engagement activities and the implementation assessments. Findings from Steps 8 and 9 were presented during a second in-person meeting with the MSWV and ON TRAC directors and representatives from the funding agencies of the GHC program. Additional data dissemination strategies were discussed (EA Step 10), including the creation of a brief report, formal presentation, and a logic model toolkit for dissemination at subsequent meetings of national stakeholders by the MSWV director and at a state-level training by members of the evaluation team.

### Data analysis

The interviews conducted to assess implementation (EA Step 8) with the directors of six past GHC recipient organizations served as the data analyzed for this project. A multi-site case study design [23] with qualitative descriptive methods was used as the approach for data analysis as it “follows an empirical method of investigation aiming to describe perceptions and experiences of the world and its phenomena.” [24], p. 2. Each local MSWV site was considered a case to form a collective case study [23]. The level of interpretation remained true to the data (i.e., an “as is” explanation) and used an embedded analysis approach – i.e., a review of case-specific attributes for each local MSWV site [23].

Qualitative analysis procedures followed three systematic steps: 1) transcript preparation, organization, and coder assignment; 2) data reduction in the form of coding and themes; and 3) data reporting in the form of figures, tables, and interpretive discussion. Interviews were recorded and transcribed for analysis. Transcriptions were crosschecked against their respective recording to ensure accuracy by the evaluation team. Transcripts were assigned by splitting the six team members evenly into two groups – three coders for physical activity-oriented projects and three coders for food-oriented projects.

**Fig. 1:**
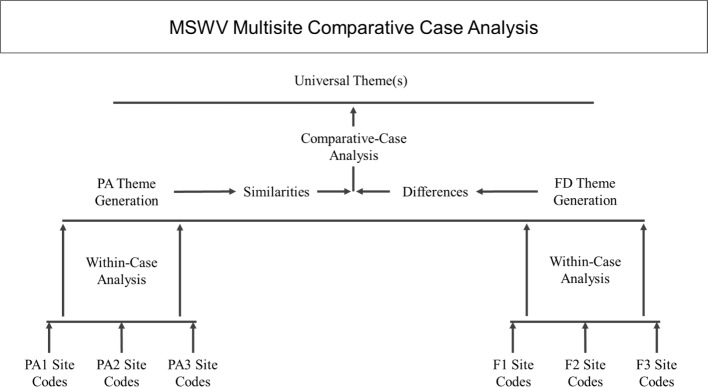
Main Street West Virginia (MSWV) Interviews Comparative Case Analysis Diagram. *Note: PA = Physical Activity-oriented project; F = Food-oriented project

Since embedded analysis often yields rich thematic findings, a comparative-case analysis [25, 26] using two cross-points (see Fig. 1) was used. First, within-case comparisons of similar codes for each project type was conducted by a leader and two coders independently coding all three transcripts by coding chunks of text using the New Comment feature in the Review tab of Microsoft® Word. The group leader then used the Combine feature in the Review tab of Microsoft® Word for each transcript to identify agreement between coders and resolve differences. Codes were combined by the two leaders to form themes and agreed upon by the coders to represent a broad and collective definition of similar transcript codes and accurate depiction of cases [27].

Secondly, a cross-case analysis was conducted using a similar process in Microsoft® Word by a single coder. Using the completed within-case analysis completed in the prior step, a final review of all six transcripts was used to generate a complete set of cross-case themes across project types. The two leaders independently reviewed this cross-case list until agreement and consensus was reached. This was done to assign common themes, highlight an overarching universal theme, and attach a unifying description for all MSWV sites.

## Results

### Within-case analysis

#### Physical activity-oriented project themes

The three MSWV sites implementing physical activity-oriented projects shared six common themes, listed in Table 3 along with representative quotes to illustrate their contextual importance. The most frequently mentioned themes were *commitment to the community*, *collaboration to complete the project*, and *limited time*. *Commitment to the community* and *collaboration to complete the project* highlight common strengths shared by the local MSWV sites with physical activity-oriented projects. *Limited time* was identified by all three local MSWV sites as an implementation barrier. *Limited time* stifled the GHC funded mini-grant projects in their ability to complete GHC application deliverables, plan for barriers (e.g., weather), and integrate measurement into their plans. However, all of these sites described how GHC mini-grant funding acted as a catalyst to stimulate projects in their communities.

**Table 3:** Themes from Interviews with Directors of Physical Activity-Oriented Projects funded by the West Virginia Growing Healthy Communities mini-grant program, 2017

Selected Theme	Quote
Commitment to the community	“Having this path there [to the depot] will provide opportunities… we are working with a group called Generation [site county name] and they want to do family fun runs…” (Physical Activity Site 2)
Collaboration to complete the project	“Healthy [site county name] is comprised of several organizations, so it’s not just the hospital and Main Street [site city name]. It’s WVU Extension, the health department, the school system. We got a couple people that are just, um, you know have their own businesses. Like a woman who has her own, her own counseling services. We have a guy who is the graphic designer, so he helps us out a lot. The library is involved. Um, Catholic Charities … we have just a couple of people that are just citizen volunteers.” (Physical Activity Site 1)
Limited time	“I’ve come to learn were never ready because every time there’s a flood, there’s a tree down, so you’re never ready. We do spring we do fall cleanup every year we go out clean up the tires, you know, they just grow [out of the ground]. This fall we’ll do another cleanup and the tires will come out again. So, it’s, it’s, amazing, we can never be ready. We can just never be ready.” (Physical Activity Site 3)
Commitment to economic stimulus	“Well I would tell you that it has increased existing business activity and that places that never or will display one or two kayaks are now displaying multiple kayaks and we see kayaks on top of cars so we know there’s activity…” (Physical Activity Site 3)
Planning barriers	“So we had over 400 people sign-up, um, and we had a good 50 or so at the kick-off and a few at the walks. I think last year was the one where the weather just got hot all a sudden so it did not go great. Yeah, so, we, you know, kept going to the group walks hoping someone would show up, but nobody really did.” (Physical Activity Site 1)
Measurement integration	“We don’t actually do that. I mean, the honest truth is health outcomes are not one of program areas. We measure economic development and that’s where all this other stuff comes in. So it’s, kind of, the, health and wellness activities contribute to economic development and that’s what we measure.” (Physical Activity Site 2)

### Food-oriented project themes

The three MSWV sites with food-oriented projects also shared six common themes. Table 4 highlights each set of themes with representative quotes to illustrate their contextual importance. The most frequently mentioned themes for the local MSWV sites with food-oriented projects were *social cohesion*, *collaboration to complete the project*, and *limited time*. *Social cohesion* and *collaboration to complete the project* highlight common strengths shared by the local MSWV sites focused on food. Through articulating their story, each site relayed how the GHC mini-grant funding encouraged social cohesion and reinforced community values. *Limited time* was identified by all three sites as a barrier to implementation. Specifically, the GHC mini-grant funding cycle limited the local sites’ ability to complete project planning, implementation, and evaluation.

**Table 4:** Themes from Interviews with Directors of Food-Oriented Projects funded by the West Virginia Growing Healthy Communities mini-grant program, 2017

Selected Theme	Quote
Social cohesion	“It is, we’re baffled at the creativity that these people come up with. It’s wonderful how they encourage each other, because they’re all there for the same reason. … It’s great to see some of the older people who’ve done this since they were children ... they assist the others and give them advice.” (Food Site 1)
Collaboration to complete the project	“We have a lot of 4H clubs, a lot of 4H clubs here, and we have WVU, of course, the master gardeners, and they’re great. They’re willing to come in when we get the high tunnels. They’re gonna come in and help the 4H clubs and help the children and teach them how to do this. So, we partner with WVU as well, we’re going to when we get the high tunnels.” (Food Site 3)
Limited time	“I think the funds arrive in February, mid to late February … and you have to expand them by the end of June. So, that doesn’t leave a lot time when we realize if other resources are needed which 9 out of 10 times that’s the case.” (Food Site 2)
Positive future direction	“We are looking at taking it one step further. I am in the process of getting ready to sit down and put together a grant for the USDA to put an actual all year-round indoor farmers market in our building. Main Street and one of the buildings in [site city name] and we are looking at putting it into one of our street level retail spots.” (Food Site 1)
Quantifying success	“Health wise we have every other week, every other two weeks or something. We have somebody coming in we offer like blood pressure, you know, that kind of thing. … It makes me happy being a grandmother myself, to see little kids walking around, with either a fresh peach rather than a cupcake or something like that.” (Food Site 3)
Measurement integration	“I don’t know whether we see that part. We haven’t really got into it because I am not an expert on the health issue. We are looking at it as an economic driver for the farms here. At one point in time there were over 1000 farmers in [site county name]… educate the low-income people … even if it is only tomatoes, or only peppers ...” (Food Site 1)

Note: a high-tunnel (or “hoophouse”) is a type of unheated greenhouse that comes in many sizes that is used to extend the growing season in farming

### Cross-case analysis

Analysis of responses from all six local MSWV sites regardless of project orientation revealed three themes: 1) c*ollaboration to complete the project,* 2) *limited time,* and 3) *measurement integration*. Each theme is presented in this narrative with representative quotes to illustrate their contextual importance. These three themes suggest a universal concept of *organizational capacity*, which is both a common strength and an area for improvement.

*Collaboration to complete the project* highlights a common strength shared by all of the local MSWV sites. Local MSWV directors discussed the importance of partnerships, program sustainability, and positive community feedback. All sites discussed plans related to a “big picture” focus of their projects. Participants displayed a deep connection to community and revitalization, exemplifying the MSA approach of “community-driven, comprehensive revitalization.” Although all descriptions varied, one quote encapsulates this concept well:

I think that Growing Healthy Communities directing [our] focus towards healthy communities, healthy life style development, is a really, really good idea. And a good way to go for West Virginia in particular, but in terms of economic development, it is what people want. (Physical Activity Site 2)

Parallel to this strength, *limited time* and *measurement integration* together were identified as barriers. Regarding time, all of the local MSWV directors expressed concern with GHC mini-grant funding timelines, project planning, and/or the mistiming of the grant cycle with food growing or construction seasons. To overcome this barrier, most sites relied on other non-profits or the local municipality for funding or personnel to extend the GHC funding cycle. This is represented in the following quote:

I think the [GHC funding] time frame should be a little longer, because in [site county name] we really haven’t started farmers market until June. That is because of the weather. So, I have to, you know, kind of figure out how to do that, but it will be carried out throughout the whole summer. Even though it will end … we will be doing it throughout the summer. (Food Site 3)

Of great importance to evaluating these mini-grants, all of the local MSWV directors expressed that *measurement integration* was an area in need of improvement. Although important, health outcome evaluation was not integrated into the grant timeline or trainings for the local organizations that were not traditionally focused on measuring health outcomes. The combined effect of funding time constraints and the organizational focus of Main Streets on economic development was that capturing outcome indicators, especially health-related ones, became secondary or was neglected because action steps for project implementation took center stage. This was not due to a lack of organizational willingness. Rather, as one interviewee noted:

“I don’t know whether we see that part we haven’t really got into it because I am not an expert on the health issue of it, we are looking at it as an economic driver…” (Food Site 1)

These cross-case themes highlight a unifying concept of MSWV sites’ *organizational capacity*. The sites’ perseverance to handle tight funding deadlines with limited organizational capacity is a testament to their passion for their communities. However, this passion can lead to personnel “burnout,” which was interlaced throughout the interviews. This suggests that MSWV programs in rural communities, especially those with only part-time staff, are tenuous and that components outside of sites’ primary focus, such as health outcome evaluation, tend to be neglected. Thus, there is a need for centralized resources and technical assistance to enhance *organizational capacity* as illustrated in the following quote:

We think that this [GHC] is amazing. To have these plans, to have these projects, and move it down the road separately. We were funded for this resource to kind a head the charge on, but this is not here yet [planned activities] and the grant is on a, you know, very short span or time-frame. So, we’re scrambling to have capacity and meet the deadlines while competing with our day jobs. It’s another project and, uh, so having all of that in the line. It would be great if we had this resource on board already … (Food Site 3)

## Discussion

The EA of the GHC mini-grant funding program accomplished the common elements of frequently used EA models [18, 20, 21], yielding key findings that are useful to public health practitioners designing mini-grant programs or involved in designing a larger robust evaluation of such programs. The first purpose of this study was to determine if this program was ready for full-scale evaluation. Findings suggest that, due to limited organizational capacity of the local Main Street organizations, tight timelines on the funding, and limited integration of health measures, an outcome evaluation was not realistic. Potential health outcomes such as physical activity or fresh fruit and vegetable consumption, generally outside the realm of the economic development focus of Main Street organizations, would logically occur *after* the grant funding period of 4–6 months which was almost exclusively focused on environment changes (e.g., building a trail, constructing community garden plots).

The second purpose of this study was to inform the implementation and evaluation of this and other programs. Due to the factors noted in the previous paragraph, any evaluation of GHC or similar mini-grants should focus on process evaluation to assess activities accomplished during the grant period, such as linear feet of trail constructed or square feet of community garden plots built. There will be a delayed effect on behavioral outcomes, such as physical activity and fruit and vegetable consumption, which could be measured in subsequent years. Critical, however, is capturing baseline outcome measures at the inception of the grant against which subsequent measures could be assessed.

Other community development entities such as MSA or the Federal Reserve Bank [28], when considering a similarly focused healthy mini-grant mechanism, may find these results particularly informative. These entities are critical for built environment change in rural U.S. communities that often lack private investment. The local MSWV organizations interviewed for this project recognized the beneficial nature of the GHC funding for community health through increasing access to healthy food and places for physical activity, echoing findings from other rural mini-grant research focused on ecological determinants of healthy behaviors in the rural U.S. [5, 6, 29, 30] The successful implementation of the funding by MSWV organizations despite barriers highlighted in the results suggests that local economic development organizations may be successful mini-grant implementation partners for public health initiatives in rural areas in addition to child care centers, non-profits, schools, health departments, and faith-based organizations evidenced in prior research [5, 13, 14]. Specifically, these economic development organizations may be valuable partners to implement rural multi-sector collaborations espoused in the National Physical Activity Plan [31] and COCOMO nutrition strategies [17]. Despite interest in health outcomes, these local organizations lacked a primary focus and experience with such outcomes, leading to an organizational focus on implementation and more familiar economic indicators. To overcome this challenge with unclear theory, program goals, and measures – which is common [18] – a funding agency should consider providing a list of outcomes, the instruments for tracking outcomes, financial resources for evaluation, and/or allow time pre-and post-implementation for measurement. Alternatively, the funding agency could hire an evaluator to work across funding sights to conduct outcome evaluation in a systematic, consistent way. Examples of this have been seen in the recent public health mini-grant literature [4–6, 32].

Despite important results, the findings should be viewed in light of limitations in the data collection and analysis methods. First, the interview responses may not capture the full extent of the requested information. Therefore, the discussion may be limited with respect to forming a fully comprehensive set of codes and themes for each MSWV site. Though applicable to MSWV, our findings may not be applicable in communities dissimilar in size or sociodemographic characteristics or to MS organizations of different staff size or structure. Findings should be interpreted with caution, as responses may be limited in scope and/or applicability based on the subjective methods used to identify the MSWV sites interviewed. However, based on the consistency found in themes and codes across different interviews, the results were deemed reliable and usable.

## Conclusions

As Schmidt [8] noted, mini-grants can facilitate community action to address health issues but accompanying support for training is critical. Our findings extend this recommendation to the topic of evaluation. While this project advanced the GHC program closer to full-scale project evaluation, there remained limited capacity among the mini-grant recipients for outcome evaluation due to a lack of resources for comprehensive evaluation and training for data collection. This limited capacity was exacerbated by the lack of municipality-specific health and economic data in rural areas. Centralized assistance for data collection and/or enhancing the local-specificity of national surveillance systems (e.g., Behavioral Risk Factor Surveillance System), especially in rural areas, are critical for overcoming this capacity limitation.

Other mini-grant funding organizations could overcome this critical barrier by dedicating resources from the moment a Call for Proposals is released to facilitate collaboration with evaluation experts to help recipients plan for, implement, and conduct outcome evaluation data collection from the outset of a grant award. Though limited, the recent public health mini-grant literature highlights the use of a central entity to evaluate the effectiveness and cost-effectiveness of multi-site, community-based mini-grant programs [4–6, 32]. If continued, this promising practice could lead to increased understanding of the health impact of rural economic development mini-grants and alleviate concerns about burnout among these organizations that are working to affect unfamiliar outcomes.

## Additional file

Additional file 1: Growing Healthy Communities Mini-grant Recipient Interview Script. (DOCX 18 kb)

## Data Availability

The datasets (i.e., transcripts) used and analyzed during the current study are available from the corresponding author on reasonable request.

## Abbreviations

DHHR: Department of Health and Human Resources
EA: Evaluability assessment
GHC: Growing Healthy Communities
MSA: Main Street America
MSWV: Main Street West Virginia
ON TRAC: Organization, Training, Revitalization, and Capacity
WV: West Virginia
