# Supplementary material for: Evaluability assessment of “growing healthy communities,” a mini-grant program to improve access to healthy foods and places for physical activity
Source: BMC Public Health. 2019 Jun 20;19:779. doi: 10.1186/s12889-019-7156-8 (PMC6585124; doi:10.1186/s12889-019-7156-8)
Supplement: Supplementary file 1 — Growing Healthy Communities Mini-grant Recipient Interview Script. (DOCX 18 kb) [file 12889_2019_7156_MOESM1_ESM.docx]

**GHC Micro-grant Recipients Evaluability Assessment**

*Interview Guide*

Introductory Script

“Thank you again for taking the time to discuss your GHC grant program today. Over the next 30-45 minutes I will be asking you a series of questions about your program. Everything we discuss will remain confidential, and I will not identify your program by name in any publications that may result from this qualitative research study. As a reminder, participation in this study is entirely voluntary and you can choose to stop at any time without penalty. This research study is being completed as part of a Doctor of Philosophy (Public Health Sciences) degree, and has been reviewed and acknowledged by the WVU Institutional Review Board.”

“Would you like to begin the interview?”

If NO: “I understand. Thank you again for letting me visit today. Have a nice day.”

If YES: “Great! To help me remember everything we discuss, I would like to audio record our interview, which will be transcribed and analyzed. Would it be okay if I audio recorded our interview today?”

IF NO: “I understand. Would it be okay if I wrote some notes during our interview?”

IF YES: “Great! Let’s get started. I’m going to turn the audio recorder on now.” [Turn on audio recorder]

| **Guiding Questions** |
| --- |
| 1. Could you tell me about your GHC program? |
| - 1. What do you find is successful about the program? What are some way you know this is happening? |
| - 1. What do you find is a challenge about the program? |
| - 1. How do you know how many people participate in ___________? |
| - 1. Is ___________ still being used? |
| - 1. How do you know ___________ is still being used? |
| - 1. What are your future plans with _______________? |
| - 1. How would go about measuring _____________? |
| 1. How do you promote ____________ in the community? In what ways do you communicate with the community? |
| 1. What health benefits do you see because of _____________? |
| 1. Do you feel like this increases the community use of downtown space ___________? |
| 1. Can you describe the champions in your community? |
| 1. Do you partner with anyone in the community? |
| 1. Can you describe these partnerships? |
| - 1. What makes these partnerships successful? |
| - 1. What makes them challenging? |

“Thank you sincerely for your time today. Once the data are analyzed, I would like to speak with you again regarding the results to get your thoughts. Have a nice day.”

Additional information to be collected from each director:

Length of time with agency

Education/Training (field, degrees, schools)

Years of Experience

Gender/Ethnicity

Skills
